# Health-related quality of life comparing standard and prolonged time-to-surgery after neoadjuvant chemoradiotherapy for esophageal cancer: results from the multicenter, randomized, controlled NeoRes II trial

**DOI:** 10.1093/dote/doag047

**Published:** 2026-05-14

**Authors:** Anders Holmén, Fahad Murad, Klara Nilsson, Ioannis Rouvelas, Mats Lindblad, Eva Szabo, Ingvar Halldestam, Ulrika Smedh, Bengt Wallner, Jan Johansson, Gjermund Johnsen, Eirik Kjus Aahlin, Hans-Olaf Johannessen, Gabriella Alexandersson von Döbeln, Geir Olav Hjortland, Naining Wang, Ying Shang, David Borg, Alexander Quaas, Isabel Bartella, Christiane Bruns, Wolfgang Schröder, Magnus Nilsson, Fredrik Klevebro, Berit Sunde

**Affiliations:** Division of Surgery and Oncology, Department of Clinical Science, Intervention and Technology, Karolinska Institutet, Stockholm, Sweden; Department of Surgery, Södersjukhuset, Stockholm, Sweden; Division of Surgery and Oncology, Department of Clinical Science, Intervention and Technology, Karolinska Institutet, Stockholm, Sweden; Department of Upper Abdominal Diseases, Karolinska University Hospital, Stockholm, Sweden; Division of Surgery and Oncology, Department of Clinical Science, Intervention and Technology, Karolinska Institutet, Stockholm, Sweden; Department of Surgery, Danderyd Hospital, Stockholm, Sweden; Division of Surgery and Oncology, Department of Clinical Science, Intervention and Technology, Karolinska Institutet, Stockholm, Sweden; Department of Upper Abdominal Diseases, Karolinska University Hospital, Stockholm, Sweden; Division of Surgery and Oncology, Department of Clinical Science, Intervention and Technology, Karolinska Institutet, Stockholm, Sweden; Department of Upper Abdominal Diseases, Karolinska University Hospital, Stockholm, Sweden; Department of Surgery, Faculty of Medicine and Health, Örebro University Hospital, Örebro, Sweden; Department of Surgery, University Hospital of Linköping, Linköping, Sweden; Department of Surgery, Sahlgrenska University Hospital, Gothenburg, Sweden; Department of Surgical and Perioperative Sciences, Surgery, Umeå University, Umeå, Sweden; Department of Surgery, Skåne University Hospital, Lund, Sweden; Department of Gastrointestinal Surgery, St. Olav’s Hospital, Trondheim University Hospital, Trondheim, Norway; Department of GI and HPB Surgery, University Hospital of Northern Norway, Tromsø, Norway and Institute of Clinical Medicine, University of Tromsø, Tromsø, Norway; Department of Gastrointestinal Surgery, Oslo University Hospital, Oslo, Norway; Division of Surgery and Oncology, Department of Clinical Science, Intervention and Technology, Karolinska Institutet, Stockholm, Sweden; Department of Radiotherapy, Karolinska University Hospital, Stockholm, Sweden; Department of Oncology, Oslo University Hospital, Oslo, Norway; Department of Clinical Pathology and Cancer Diagnostics, Karolinska University Hospital, Stockholm, Sweden; Department of Medicine Huddinge, Karolinska Institutet, Stockholm, Sweden; Department of Oncology, Skåne University Hospital, Lund, Sweden; Institute of Pathology, University of Cologne, Cologne, Germany; Department of General, Visceral, Cancer and Transplantation Surgery, University Hospital of Cologne, Cologne, Germany; Department of General, Visceral, Cancer and Transplantation Surgery, University Hospital of Cologne, Cologne, Germany; Department of General, Visceral, Cancer and Transplantation Surgery, University Hospital of Cologne, Cologne, Germany; Division of Surgery and Oncology, Department of Clinical Science, Intervention and Technology, Karolinska Institutet, Stockholm, Sweden; Department of Upper Abdominal Diseases, Karolinska University Hospital, Stockholm, Sweden; Division of Surgery and Oncology, Department of Clinical Science, Intervention and Technology, Karolinska Institutet, Stockholm, Sweden; Department of Upper Abdominal Diseases, Karolinska University Hospital, Stockholm, Sweden; Division of Surgery and Oncology, Department of Clinical Science, Intervention and Technology, Karolinska Institutet, Stockholm, Sweden; Department of Upper Abdominal Diseases, Karolinska University Hospital, Stockholm, Sweden

**Keywords:** esophageal cancer, health-related quality of life, neoadjuvant chemoradiotherapy, time-to-surgery

## Abstract

Standard time-to-surgery after neoadjuvant chemoradiotherapy for esophageal cancer has historically been 4–6 weeks. Observational studies have suggested improved oncological outcomes and health-related quality of life in patients after prolonged time-to-surgery. This study aimed to investigate whether prolonged time-to-surgery is associated with improved health-related quality of life compared to the standard interval. This study is a secondary endpoint analysis within the NeoRes II trial, in which patients with locally advanced resectable esophageal cancer were randomized to either standard time-to-surgery (4–6 weeks) or prolonged time-to-surgery (10–12 weeks). The primary endpoints have been reported previously. Health-related quality of life was assessed using the EORTC QLQ-C30 and QLQ-OG25 questionnaires at time of diagnosis, preoperatively, and at 6-months and 1–5 years postoperatively. A total of 249 patients were randomized of whom 192 were available for health-related quality of life analyses, with 97 (51%) assigned to standard time-to-surgery and 95 (49%) to prolonged time-to-surgery. The groups were well-matched regarding baseline characteristics. Preoperatively, within a week of the day of surgery, patients in the prolonged time-to-surgery group reported better global quality of life, improved physical functioning, and less fatigue, coughing, dysphagia, odynophagia, pain, discomfort, and weight loss compared to those in the standard time-to-surgery group. These benefits were observed at 6 months postoperatively, but not thereafter during follow-up, where no significant differences were observed. Although prolonged time-to-surgery was associated with better preoperative health-related quality of life, no such benefits were observed postoperatively. In addition, the results from the primary endpoint analysis suggested that longer time-to-surgery was associated with worse oncological outcomes. This, together with the results of the current sub-study, strongly supports that the standard time-to-surgery of 4–6 weeks should be recommended after neoadjuvant chemoradiotherapy for esophageal cancer.

## INTRODUCTION

Timing of surgery after neoadjuvant treatment for patients with esophageal cancer has been a debated topic, largely due to absence of randomized data concerning ideal time-to-surgery (TTS), with only observational data, prone to selection bias, previously available. Since the 1960s, the standard TTS has been 4–6 weeks, initially based on a single-center study that concluded TTS of 6 weeks allowed patients to recover from neoadjuvant therapy while local inflammation subsides, and not leading to tumor progression.^1^ In the decades since, there has been substantial development in neoadjuvant therapy, and current treatment differs considerably from 60 years ago.

Current guidelines suggest different treatment strategies depending on esophageal cancer histological subtypes: For squamous cell carcinoma the recommendation is neoadjuvant chemoradiotherapy (nCRT) or definitive chemoradiotherapy (dCRT); For adenocarcinoma, the recommendation perioperative chemotherapy, or if not tolerated by the patient, nCRT.^2^

In recent years, the optimal TTS has been questioned. While some studies suggested an association between prolonged TTS and increased rate of R0 resection, improved survival, and health-related quality of life (HRQL), others indicated no survival benefit at all.^3–6^ The NeoRes II-trial evaluated complete histologic response as primary endpoint in patients with adenocarcinoma, which has been presented in detail. In conclusion, the study failed to show any significant differences in short-term outcomes concerning TTS regarding histologic response, postoperative morbidity, or for complications such as anastomotic leak, conduit necrosis, chyle leak, pneumonia, or respiratory failure.^6^ In addition, although not statistically significant, there was a trend toward worse overall survival in the prolonged TTS-group, with a statistically significant difference in the subgroup of patients with poor pathological response to nCRT.^5,6^

Before the NeoRes II data were published, there was a gradual shift in clinical practice toward increased TTS after neoadjuvant treatment. Data from the Swedish National Register for Esophageal and Gastric cancer show that between January 2006 and April 2017 approximately half the patients underwent surgery after 7 weeks or longer after completed neoadjuvant treatment.^5^ How a prolonged TTS, after preoperative neoadjuvant oncological treatment affects patients HRQL after esophagectomy, has still not previously been reported.

The aim of this study was to evaluate the impact of prolonged TTS of 10–12 weeks on pre- and postoperative HRQL compared to the standard TTS of 4–6 weeks after nCRT. The hypothesis is that a prolonged TTS will lead to improved recovery and HRQL pre- and postoperatively.

## METHODS

### Study design

**Fig. 1:**
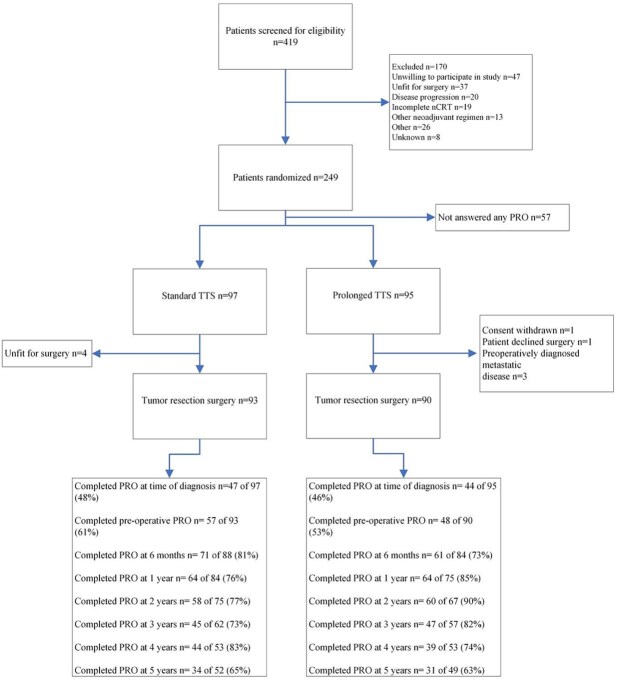
CONSORT diagram for health-related quality-of-life assessment in the study.

The details of this trial have been described in previous publications.^5,6^ Patients were randomized to two different exposures, either standard TTS (4–6 weeks) or prolonged TTS (10–12 weeks) following standard CROSS type nCRT. HRQL data was prospectively collected using the validated European Organization for Research and Treatment of Cancer (EORTC) questionnaires Core Quality of Life questionnaire (QLQ-C30) and Esophago-gastric quality of life questionnaire (QLQ-OG25) as patient reported outcomes at time of diagnosis, preoperatively after completed nCRT, postoperatively at 6 months, and at 1, 2, 3, 4, and 5 years after surgery. Patients were followed-up for up to 5 years after surgery, until death or end of follow-up. (Fig. 1).

### Study cohort

Inclusion and exclusion criteria have previously been published.^5,6^ All patients who fulfilled the inclusion criteria at the time of diagnosis were invited to participate in the trial. Randomization was performed after completed nCRT.

Between February 2015 and March 2019, 249 patients were randomized at 10 participating European university hospitals (6 in Sweden, 3 in Norway, and 1 in Germany: Karolinska University Hospital, Sahlgrenska University Hospital, Akademiska University Hospital, Skåne University Hospital, Linköping University Hospital, Örebro University Hospital, Norrland’s University Hospital, Oslo University Hospital, St. Olavs Hospital, Trondheim University Hospital, University Hospital in Northern Norway, and University Hospital of Cologne).

### Study questionnaires

#### EORTC-QLQ-C30 (version 3)

The EORTC QLQ-C30 consists of five functional scales (physical, role, emotional, cognitive, and social) and one global health/quality of life scale, three symptom scales (fatigue, nausea and vomiting, and pain) and six single items (dyspnea, insomnia, loss of appetite, constipation, diarrhea, and financial difficulty). The EORTC QLQ-C30 has been validated for measuring quality of life in patients in oncological trials.^7^ In the questionnaire patients are asked about their level of activity, ability to go on short or long walks, their ability to perform activities of daily living, and measurements of different functions such as social, emotional, physical, and role functioning. They are also asked about pain, shortness of breath, sleep quality and insomnia, nausea, vomiting, constipation, anxiety, memory, and a global assessment of health and life-quality through different questions to assess their general HRQL.

#### EORTC-QLQ-OG-25

The questionnaire QLQ-OG25 measures disease specific metrics regarding the HRQL of patients with esophageal, gastric, or gastroesophageal junction cancer. The questionnaire evaluates symptoms such as dysphagia, eating restrictions, reflux, odynophagia, anxiety, and pain. QLQ-OG25 is recommended as a supplement to the QLQC30 questionnaire when assessing HRQL in patients with esophageal-, gastroesophageal junction-, or gastric cancer.^8^

#### Interpretation of questionnaires

The questions in the QLQ-C30 and QLQ-OG25 all have four response alternatives (1, not at all; 2, a little; 3, quite a bit; 4, very much), except for two questions in the C30 regarding overall health and QOL assessment, where there are seven response alternatives ranging from very poor to excellent. The answers is converted to a scoring system by calculating the average of each individual item that contribute to a scale, outputting a raw score. A linear transformation model is used on the raw score to calculate a final score ranging from 0 to 100. A higher score in the symptom scales indicates a higher symptom burden, while a higher score in the functioning scales indicates better functioning.^9^

### Statistical analysis

Statistical analyses were performed using Stata® software version 16.0 (StataCorp, College Station, Texas, USA) and IBM SPSS statistics for Macintosh, version 29.0.2.0 (IBM Corp, Armonk, New York, USA). All analyses followed the intention-to-treat principle. Descriptive statistics were used to summarize baseline characteristics. Categorical data were presented as numbers and percentages, while numerical data were presented as means and mean differences (MDs). Data from the QLQ-C30 and QLQ-OG25 were linearly converted to a 0–100 scale according to the EORTC scoring manual.^9^ Analyses were conducted to compare the two treatment groups by calculating means and MDs using linear regression models with 95% confidence intervals. A MD of at least 10 points was defined as a clinically significant difference,^10^ and statistical significance was set at *P* < 0.05. A sensitivity analysis was performed to study the characteristics of patients who responded to the questionnaires to assess whether they differed over time between the groups.

### Ethical approval

The study was approved by the Research Ethics Committees in Sweden (Regionala etikprövningsnämnden in Stockholm, approval numbers: 2014/748-31, 2015/1271-32, 2016/626-32), Norway (REK Sør-Øst 2014/1938) and the Institutional Review Board of the University of Cologne, Cologne, Germany (IRB approval number: 17-012). The study was registered in ClinicalTrials.gov (NCT02415101).

## RESULTS

A total of 249 patients were included and randomized in the NeoRes II trial. Of these, 125 patients were randomized to standard TTS, and 124 patients to prolonged TTS. Out of the included 249 patients, 192 (77%) patients answered one or more HRQL questionnaires and were included in this study. Among these, 97 (51%) patients were in the standard TTS group, and 95 (49%) in the prolonged TTS group. Mean age was similar, 65 years in the standard TTS group versus 63 years in the prolonged TTS group, and a majority of patients (83% vs. 86%, standard TTS vs. prolonged TTS) were male. There were some minor differences between the groups regarding comorbidities: cardiovascular (36% vs. 25.3%), pulmonary (10.3% vs. 6.3%), and diabetes (11.3% vs. 14.7%). Tumor stage was similar between the groups both at clinical and pathological evaluation. Mean time to surgery from completed nCRT in the standard TTS group was 39 days (range: 26–75), and in the prolonged TTS group 74 days (range: 34–109) (Table 1).

**Table 1:** Baseline characteristics of all patients who answered one or more HRQL questionnaire

Number (percent)	Standard TTS	Prolonged TTS
Total	97 (50.6)	95 (49.4)
Age, mean (range)	65 (34–78)	63 (42–78)
Sex		
Male	80 (82.5)	82 (86.3)
Female	16 (16.5)	12 (12.6)
Missing	1 (1.0)	1 (1.1)
Comorbidity		
Diabetes	11 (11.3)	14 (14.7)
Cardiovascular	36 (37.1)	24 (25.3)
Pulmonary	10 (10.3)	6 (6.3)
Smoking	30 (30.9)	32 (33.7)
Alcohol	3 (3.1)	4 (4.2)
Histology		
Adenocarcinoma	77 (79.4)	73 (76.8)
Squamous cell carcinoma	19 (19.6)	21 (22.1)
Missing	1 (1.0)	1 (1.1)
Tumor stage		
1	1 (1.0)	0 (0)
2	22 (22.7)	26 (27.4)
3	60 (61.9)	56 (58.9)
4	13 (13.4)	12 (12.6)
Missing	1 (1.0)	1 (1.1)
Nodal stage		
0	45 (46.4)	34 (35.8)
1	37 (38.1)	42 (44.2)
2	11 (11.3)	14 (14.7)
3	3 (3.1)	4 (4.2)
Missing	1 (1.0)	1 (1.1)
Time to surgery, mean (range)	39 (26–75)	74 (34–109)

HRQL—Health-related quality of life, TTS—time-to-surgery.

### HRQL assessment

At time of diagnosis, the patient-reported outcomes (PRO) were completed by 47/97 (48%) of patients in the standard TTS group, and 44/95 (46%) of the patients in the prolonged TTS group. In the standard TTS group, 93/97 (96%) patients underwent surgery and 57/93 (61%) reported preoperative PRO. In the prolonged TTS group, 90/95 (95%) patients underwent surgery and 48/90 (53%) reported the preoperative PRO. The 6-month postoperative follow-up was completed by 71/88 (81%) in the standard TTS group, and 61/84 (73%) in the prolonged TTS group. The response rates over the long term follow-ups varied, as can be seen in the consort diagram (Fig. 1).

### HRQL

- **Time of diagnosis**

No clinically or statistically significant differences were seen between the two groups at time of diagnosis concerning HRQL. Most commonly occurring symptoms were dysphagia, problems with eating, odynophagia, and anxiety (Table 2).

- **Preoperative after completed nCRT**

**Table 2:** Health-related quality of life at time of diagnosis comparing standard and prolonged time-to-surgery

	Standard TTS	Prolonged TTS	Mean difference	
	Mean score	Mean score	MD (95% CI)	*P*-value
**QLQ-C30**				
Global QoL	66	68	2 (−6, 10)	0.644
**Functional status**				
Physical functioning	87	92	5 (−1, 9)	0.099
Role functioning	82	81	−1 (−1, 9)	0.782
Emotional functioning	76	74	−2 (−9, 5)	0.590
Cognitive functioning	91	86	−5 (−11, 2)	0.159
Social functioning	83	78	−5 (−14, 4)	0.282
**Symptom scales**				
Fatigue	23	28	5 (−4, 13)	0.310
Nausea/Vomiting	15	10	−5 (−13, 2)	0.122
Pain	22	19	−3 (−12, 6)	0.532
Dyspnea	18	18	0 (−8, 9)	0.896
Insomnia	25	25	0 (−10, 10)	0.951
Appetite loss	25	24	−1 (−13, 10)	0.802
Constipation	14	14	0 (−10, 10)	0.968
Diarrhea	16	9	−7 (−15, 1)	0.098
Financial difficulty	6	5	−1 (−7, 6)	0.870
Body image	92	86	−6 (−15, 2)	0.160
**QLQ-OG25**				
**Symptom scales**				
Dysphagia	27	25	−2 (−11, 8)	0.789
Problems with eating	34	38	4 (−7, 15)	0.483
Reflux	12	9	−3 (−10, 4)	0.395
Odynophagia	30	34	4 (−6, 16)	0.413
Pain and discomfort	23	22	−1 (−11, 9)	0.833
Anxiety	59	58	−1 (−12, 9)	0.783
Eating with others	24	19	−5 (−17, 7)	0.434
Dry mouth	16	16	0 (−10, 9)	0.912
Trouble with taste	9	15	6 (−3, 15)	0.167
Trouble swallowing saliva	8	12	4 (−5, 12)	0.379
Choked when swallowing	18	20	2 (−10, 13)	0.765
Trouble with coughing	29	24	−5 (−14, 3)	0.229
Trouble talking scale	4	2	−2 (−6, 1)	0.177
Weight loss scale	18	18	0 (−10, 11)	0.956
Hair loss scale	17	27	10 (−46, 66)	0.685

HRQL—health-related quality of life, QLQ-C30—Core Quality of Life questionnaire, QLQ-OG25—Esophago-gastric quality of life questionnaire, TTS—time-to-surgery.

After nCRT, shortly before surgery, patients in the prolonged TTS group reported better Global QoL (MD = 13, *P* < 0.001), and better Physical functioning (MD = 8, *P* = 0.002), although the latter was not clinically significant.

Patients in the prolonged TTS group reported both clinically and statistically less symptoms regarding fatigue (MD = 14, *P* **<** 0.001), pain (MD = 12 *P* = 0.008), dyspnea (MD = 12, *P* = 0.008), appetite loss (MD = 17, *P* = 0.001), problems with eating (MD = 14, *P* = 0.003), odynophagia (MD = 11, *P* = 0.025), pain and discomfort (MD = 14, *P* **<** 0.001), trouble with taste (MD = 11, *P* **=** 0.028), and weight loss (MD = 18, *P* **<** 0.001) (Table 3).

- **Postoperative follow-ups**

**Table 3:** Patient-reported outcome measurements preoperatively comparing standard and prolonged time-to-surgery

	Standard TTS	Prolonged TTS	Standard vs. Prolonged	
	Mean score	Mean score	MD (95% CI)	*P*-value
**QLQ-C30**				
Global QoL	61	74	13 (6, 20)	**<0.001**
**Functional status**				
Physical functioning	80	88	8 (3, 14)	**0.002**
Role functioning	67	76	9 (0, 19)	**0.041**
Emotional functioning	75	80	5 (−2, 12)	0.159
Cognitive functioning	88	90	2 (−3, 8)	0.406
Social functioning	73	78	5 (−3, 14)	0.212
**Symptom scales**				
Fatigue	39	25	−14 (−21, 6)	**<0.001**
Nausea/Vomiting	15	9	−6 (−12, 1)	0.072
Pain	25	13	−12 (−20, −3)	**0.008**
Dyspnea	31	19	−12 (−20, −4)	**0.005**
Insomnia	29	21	−8 (−18, 0)	0.052
Appetite loss	32	15	−17 (−27, −7)	**0.001**
Constipation	20	16	−4 (−13, 5)	0.382
Diarrhea	14	9	−5 (−12, 2)	0.198
Financial difficulty	13	6	−7 (−13, 1)	0.099
Body image	79	85	6 (−2, 15)	0.129
**QLQ-OG25**				
**Symptom scales**				
Dysphagia	19	10	−9 (−17, −2)	**0.009**
Problems with eating	34	20	−14 (−23, −5)	**0.003**
Reflux	14	9	−5 (−11, 1)	0.123
Odynophagia	25	14	−11 (−20, −1)	**0.025**
Pain and discomfort	26	12	−14 (−22, −7)	**<0.001**
Anxiety	55	53	−2 (−12, 7)	0.595
Eating with others	17	11	−6 (−15, 4)	0.229
Dry mouth	22	17	−5 (−14, 4)	0.253
Trouble with taste	26	15	−11 (−21, −1)	**0.028**
Trouble swallowing saliva	12	5	−7 (−14, −2)	**0.010**
Choked when swallowing	12	11	−1 (−9, 5)	0.624
Trouble with coughing	30	21	−9 (−16, −1)	**0.027**
Trouble talking scale	4	3	−1 (−6, 3)	0.539
Weight loss scale	30	12	−18 (−27, −9)	**<0.001**
Hair loss scale	28	16	−12 (−29, 4)	0.148

Bold numbers are used to signify a statistically significant p-value >0.05. HRQL—health-related quality of life, QLQ-C30—Core Quality of Life questionnaire, QLQ-OG25—Esophago-gastric quality of life questionnaire, TTS—time-to-surgery.

At the 6-month follow-up after surgery, patients in the prolonged TTS group reported clinically and statistically less symptoms regarding insomnia (MD = 11, *P* = 0.024), constipation (MD = 10, *P* = 0.029), problems with eating (MD = 10, *P* = 0.023). During subsequent years following surgery there was no clinically relevant differences at all postoperative PRO measurements (Tables 4 and 5).

**Table 4:** Patient-reported outcome measurements 6 months postoperatively comparing standard and prolonged time-to-surgery

	Standard TTS	Prolonged TTS	Standard vs. Prolonged	
	Mean score	Mean score	MD (95% CI)	*P*-value
**QLQ-C30**				
Global QoL	63	68	5 (−1, 12)	0.121
**Functional status**				
Physical functioning	76	80	4 (−2, 10)	0.221
Role functioning	63	70	7 (−3, 17)	0.183
Emotional functioning	77	83	6 (−1, 13)	0.093
Cognitive functioning	84	86	2 (−5, 9)	0.551
Social functioning	68	74	6 (−3, 15)	0.186
**Symptom scales**				
Fatigue	39	32	−7 (−14, 0)	0.058
Nausea/Vomiting	18	14	−4 (−19, 2)	0.209
Pain	29	20	−9 (−17, 0)	**0.042**
Dyspnea	35	32	−3 (−12, 7)	0.552
Insomnia	29	18	−11 (−20, −1)	**0.024**
Appetite loss	31	22	−9 (−19, 2)	0.102
Constipation	22	12	−10 (−19, −1)	**0.029**
Diarrhea	24	19	−5 (−15, 5)	0.269
Financial difficulty	14	11	−3 (−10, 5)	0.530
Body image	77	83	6 (−3, 16)	0.172
**QLQ-OG25**				
**Symptom scales**				
Dysphagia	18	13	−5 (−12, 1)	0.109
Problems with eating	40	30	−10 (−18, −1)	**0.023**
Reflux	21	17	−4 (−11, 3)	0.259
Odynophagia	18	16	−2 (−9, 3)	0.431
Pain and discomfort	27	28	1 (−8, 9)	0.864
Anxiety	48	39	−9 (−18, 2)	0.110
Eating with others	17	12	−5 (−13, 4)	0.290
Dry mouth	28	18	−10 (−20, −1)	**0.030**
Trouble with taste	25	18	−7 (−17, 2)	0.136
Trouble swallowing saliva	10	6	−4 (−9, 3)	0.306
Choked when swallowing	17	14	−3 (−10, 6)	0.581
Trouble with coughing	42	33	−9 (−18, 0)	0.039
Trouble talking scale	7	7	0 (−6, 5)	0.923
Weight loss scale	40	28	−12 (−24, −1)	**0.032**
Hair loss scale	23	9	−14 (−37, 9)	0.234

Bold numbers are used to signify a statistically significant p-value >0.05. HRQL—health-related quality of life, QLQ-C30—Core Quality of Life questionnaire, QLQ-OG25—Esophago-gastric quality of life questionnaire, TTS—time-to-surgery.

**Table 5:** Patient-reported outcome measurements 5 years postoperatively comparing standard and prolonged time-to-surgery

	Standard TTS	Prolonged TTS	Standard vs. Prolonged	
	Mean score	Mean score	MD (95% CI)	*P*-value
**QLQ-C30**				
Global QoL	72	78	6 (−5, 18)	0.282
**Functional status**				
Physical functioning	82	88	5 (−4, 15)	0.229
Role functioning	73	80	7 (−9, 24)	0.391
Emotional functioning	85	88	3 (−8, 15)	0.578
Cognitive functioning	87	86	0 (−10, 10)	0.931
Social functioning	78	76	−2 (−18, 14)	0.815
**Symptom scales**				
Fatigue	28	30	2 (−12, 17)	0.737
Nausea/Vomiting	13	13	0 (−13, 12)	0.964
Pain	24	13	−11 (−26, 4)	0.145
Dyspnea	25	30	5 (−10, 20)	0.504
Insomnia	20	12	−8 (−23, 6)	0.245
Appetite loss	17	16	−1 (−18, 15)	0.863
Constipation	19	10	−9 (−23, 6)	0.254
Diarrhea	24	13	−11 (−26, 4)	0.141
Financial difficulty	3	9	6 (−5, 17)	0.267
Body image	85	90	5 (−10, 20)	0.543
**QLQ-OG25**				
**Symptom scales**				
Dysphagia	12	11	−1 (−13, 11)	0.825
Problems with eating	26	23	−2 (−18, 13)	0.744
Reflux	20	27	7 (−9, 23)	0.400
Odynophagia	13	12	−1 (−14, 12)	0.876
Pain and discomfort	25	17	−9 (−26, 8)	0.315
Anxiety	27	21	−6 (−22, 9)	0.421
Eating with others	17	13	−4 (−22, 15)	0.693
Dry mouth	20	10	−10 (−25, 5)	0.187
Trouble with taste	9	3	−6 (−14, 1)	0.090
Trouble swallowing saliva	3	1	−1 (−6, 3)	0.611
Choked when swallowing	11	12	1 (−10, 13)	0.807
Trouble with coughing	23	27	5 (−8, 17)	0.467
Trouble talking scale	3	6	3 (−4, 11)	0.386
Weight loss scale	16	15	−1 (−17, 16)	0.918
Hair loss scale	20	33	13 (−42, 69)	0.541

HRQL—health-related quality of life, QLQ-C30—Core Quality of Life questionnaire, QLQ-OG25—Esophago-gastric quality of life questionnaire, TTS—time-to-surgery.

### Sensitivity analysis

To evaluate the robustness of our data and examine potential confounding concerning patients’ willingness to undertake the HRQL assessments, a sensitivity analysis was performed comparing the characteristics of the patients who answered the HRQL questionnaires with those that did not. This was performed at each specific time point with regards to sex, age, histologic subtype, TNM score, and Clavien–Dindo score. We found no statistically significant differences between patients who took part of the HRQL assessments, with those that did not.

## DISCUSSION

The results of the current study suggest no long-term differences, except for 6 months postoperatively, regarding HRQL depending on TTS. In the time span between nCRT and surgery, there was a decline in HRQL in both groups, although less pronounced in patients with prolonged TTS, suggesting that longer recovery after nCRT may be beneficial concerning HRQL at the time of surgery. However, these advantages did not translate into better postoperative HRQL, as no differences in HRQL between the allocated TTS groups were detected in the postoperative measurements. Since data from the NeoRes II trial suggest worse oncological outcomes in patients with a prolonged TTS, this sub-study further supports adhering to the standard TTS practice after nCRT in the treatment of esophageal and gastroesophageal junction cancer.

Previous research on the effect of TTS on HRQL in patients with esophageal cancer is limited, but deterioration of HRQL after nCRT is a well-known problem. The negative impact of nCRT on HRQL align with what has been described by Noordman *et al.*, reporting a decline in all HRQL-parameters during, and two weeks after nCRT, with physical functioning and odynophagia returning to baseline levels after eight and four weeks respectively.^11^ Similarly, Van Meerten *et al.* has described that nCRT causes considerable deterioration in most aspects of HRQL, with gradual improvements observed within 4–8 weeks following nCRT.^12^ Other studies have reported a similar tendency.^13,14^ This is in line with our finding of better HRQL observed in the prolonged compared to standard TTS group measured just before surgery, as patients had more time to recover from the side effects of nCRT in the prolonged TS group.

Strengths of the study include the randomized controlled design with NeoRes II being the only RCT to date, comparing standard TTS to prolonged TTS after nCRT in patients with esophageal or gastroesophageal junction cancer. Long-term follow-up of 5 years allows for investigation of long-term complications, survival and HRQL after prolonged compared to standard TTS.^6,15^ The HRQL assessments, using the EORTC QLQ-C30 and QLQ-OG25 questionnaires, which are validated and considered highly reliable for reporting HRQL in patients with esophageal and gastro-esophageal junction cancer, presents another strength.^8,16,17^

The trial has some weaknesses that have been discussed in prior publications.^6,15^ Among those mentioned was that not all patients were operated on in accordance with the protocol. In eight patients (7%) surgery was delayed >7 weeks in the standard TTS group, mainly because not considered appropriately recovered after nCRT. But, since this represents only a small proportion of patients who were managed in accordance with how they would be treated in normal clinical settings, this was suggested to have a negligible impact on the external validity. Another weakness of the current study was the uneven distribution of the HRQL questionnaires among the participating centers, limiting the number of patients that were able to respond. The reported response rates did not include patients who had died, or were not contacted because of logistical capacities. As a result of this, the response rates were high as these patients were not included in the denominator. Sensitivity analysis did, however, fail to show any differences in patient characteristics between those who responded and those who did not. However, the analysis did not account for completion of nCRT, and therefore complications related to chemoradiotherapy were not assessed for, which could explain the improvement of HRQL in the prolonged TTS group. Finally, the open-label design presents another weakness as it introduces a potential risk of bias.

A weakness not unique to our study, nor to the studies by Noordman and Van Meerten, but indeed pervasive in the field of esophageal cancer, is the mix of cancer subtypes in the clinical studies. Since squamous cell carcinoma and adenocarcinoma differ significantly in etiology, demographics, treatment strategies, and prognosis, it is inappropriate to study them as a single disease entity. Patients with squamous cell carcinoma are also more frequently treated with radiotherapy, known to cause complications such as esophagitis, pneumonitis, and cardiac issues further linked to dysphagia, odynophagia, coughing problems, and dyspnea, all inflicting patients HRQL.^18^ The issue of combining different cancer subtypes introduces multiple sources of bias and increases the risk of drawing incorrect conclusions. This is, without question, a significant and persistent problem in the field of esophageal cancer research.

## CONCLUSION

The results from this sub-study suggest that there were no significant long-term benefits with prolonged time to surgery in regard to HRQL. This, together with the results of primary endpoint analysis that demonstrated that longer TTS was associated with worse oncological outcomes suggests that the standard TTS of 4–6 weeks should be recommended after nCRT for esophageal cancer.

## ABBREVIATIONS

CI, confidence interval; CROSS, chemoradiotherapy for esophageal cancer followed by surgery; CT, chemotherapy; dCRT, definitive chemoradiotherapy; ECOG, Eastern cooperative oncology group; EORTC, European Organization for Research and Treatment of Cancer; Gy, Gray; HRQL, health-related quality of life; MD, mean difference; nCRT, neoadjuvant chemoradiotherapy; nCT, neoadjuvant chemotherapy; pCT, perioperative chemotherapy; PRO, patient-reported outcomes; QLQ-C30, core quality of life questionnaire; QLQ-OG25, esophago-gastric quality of life questionnaire; RT, radiotherapy; TTS, time-to-surgery

## Notes

Financial support: None.

Potential competing interests: None.
